# “[T]he most precise and thorough understanding of the situation we are struggling to change”: re-capturing emancipatory disability research

**DOI:** 10.3389/fsoc.2025.1562498

**Published:** 2025-04-23

**Authors:** Luke Beesley

**Affiliations:** Department of Politics, The University of Manchester, Manchester, United Kingdom

**Keywords:** emancipatory disability research, disability activism, scientific method, research democratization, credible commitment problem

## Abstract

This article seeks to contribute to a refoundation of the analytic, qualitative and quantitative methods associated with Emancipatory Disability Research (EDR)—an episto-political approach to disability research which places lay disabled people in positions of authority over research design, operation, and analysis of projects undertaken by professional academics. The argument of this article is that a significant reason for EDR's meager impact on political practice, the burnout and disillusionment of some of its most talented proponents, and its failure to develop beyond limited applications in sociology and disability studies lies in the disjointed and asymmetrical development of its aims and methods. I indicate, particularly, that the core evaluation signifiers for EDR's success (that disabled people concretely benefit from the research, and control both its future direction and the uses made of it) rested on an initial demand from disabled activists for scientific rigor and a realist ontology in research which were subsequently rejected by EDR's academic advocates. Without a grounding in the scientific method, a meta-theory of subject-object relations and knowledge, or an evaluative framework for the objective accuracy of input concepts; EDR's research framework prevented practitioners from producing outputs for which there was a demonstrable demand, while promising forms of research for which there was not.

## 1 Introduction

This article seeks to contribute to a refoundation of the analytic, qualitative and quantitative methods associated with Emancipatory Disability Research (EDR)— an episto-political approach to disability research which places lay disabled people in positions of authority over research design, operation, and analysis of projects undertaken by professional academics. The argument of this article is that a significant reason for EDR's meager impact on political practice, the burnout and disillusionment of some of its most talented proponents, and its failure to develop beyond limited applications in sociology and disability studies lies in the disjointed and asymmetrical development of its aims and methods. I indicate, particularly, that the core evaluation signifiers for EDR's success (that disabled people concretely benefit from the research, and control both its future direction and the uses made of it) rested on an initial demand from disabled activists for scientific rigor and a realist ontology in research which were subsequently rejected by EDR's academic advocates. Without a grounding in the scientific method, a meta-theory of subject-object relations and knowledge, or an evaluative framework for the objective accuracy of input concepts; EDR's research framework prevented practitioners from producing outputs for which there was a demonstrable demand, while promising forms of research for which there was not.

EDR was proposed in the early 1990s by (mostly) disabled academics in Britain and the Irish Republic who were involved in or sympathetic to Disabled People's Movements (DPMs) in those countries^1^. Almost universally coming to disability politics after beginning their research careers in sociology and social policy, EDR's progenitors came to see the prevalent research practice of their fields as a constituent parts of a disabling society, rather than motors of social change. So long as research was controlled and disseminated by unaccountable academics, these thinkers argued, it was bound to reproduce the social prejudices and material inequality of the institutions which birthed it—including their exclusionary and condescending attitudes to marginalized groups, and assumptions that social or welfare policy is the preserve of elites.^2^

Their response was to invert the social relationship underpinning academic research. EDR proposed a rigorous set of research principles to address the inconsistent preferences of researchers and disabled participants' previously limited influence over their behavior. At its core, it made disabled people's co-operation with research conditional on researchers abiding by six epistemological, ontological, and methodological principles:

1. The use of the “social model of disability” (i.e., the claim that disability is socially, rather than biologically caused) as the theoretical basis for research.
2. Surrendering claims to objectivity in favor of participants and movement actors emancipatory political commitments
3. The focusing of research topics and project design around outcomes which will bring either practical material benefit to disabled people, or empower them to remove disabling barriers
4. The devolution of power over research planning and decision-making to disabled people to ensure maximal accountability of researchers to their subjects and/or the DPM.
5. A commitment to describe participants' personal experience of disablement without distortion, while representing it as part of a collective experience of oppression.
6. The selection of research methods to reflect the preferences and priorities of participants and the DPM (Stone and Priestley, 1996, p.706).

Of these principles, decentralization of power over how research is planned, conducted and evaluated is the most central—it is the accountability arising from this which safeguards the appropriateness of methods, theoretical groundings, research topics, representation of participants and empowering outcomes implied by the other five. It is grounded in a recognition that researchers do not come to disabled people as autonomous moral and ethical agents; but as conditioned by and dependent on structures which are divorced from disabled people's interests, priorities, and welfare. Academics are compelled to write papers acceptable in style and content to their colleagues—not disabled people more generally—to be successful in their field; and to make recommendations that are (plausibly) amenable to governments, firms, and state agencies—not necessarily disabled people—to influence social policy.

Given these conflicting incentive structures “disabled people and their organizations should be wary of researchers,” and attempt to make them equally dependent on disabled participants or movement agents (Barnes, 1996a, p. 107). Operationally, devolution should, wherever possible, take the form of a supervisory group of disabled participants and/or movement representatives empowered by the research agreement to make binding decisions about the research's aims, structure, and activities at each stage of its design, implementation, and dissemination. If researchers fail to follow its instructions, the exit of the supervisory group would effectively negate consent agreements with all participants. Where this is not possible, any participant's consent should, minimally, be made conditional on their approval of finished reports or papers (Barnes, 1992, p. 122-3).

This article argues that the failure of emancipatory research to generalize was primarily the result of a problem in its negotiating framework – not the moral or epistemological foibles of its academic proponents, the hostility of its opponents, or its funding environment (as other scholars have argued). The second of its six principles obliges all interested parties to sacrifice claims to objectivity, while the fifth focuses researchers' attention on the subjective experience of disablement as their primary data point. Given that potential participants already know very well what *their* subjective experience is, and DPM actors are likely to recognize it from their political and community building work, EDR's attractions to them are modest. Emancipatory research solves the problem of researchers' commitment, but at the expense of committing everyone involved to findings which add little to disabled partners' projects of self-empowerment or social transformation. The incentives of non-academics to give their time and effort to it, then, are minimal.

This hypothesis is not so much new as it is previously underdeveloped. Finkelstein (1998, p. 860) pointed out forcefully in a short book review that a focus on the personal experience of oppression “uncover[s] little more than the known debilitating effects of living in a world designed for people with abilities.” Without turning their attention to the objective dynamics and structures of the social world which cause disabled people's disadvantage, emancipatory researchers make a fetish of participants' control of research practice while ignoring that they already know (and thus already control) the knowledge under discussion. Similarly, during Mike Oliver's (1997, p. 84–5) disengagement from emancipatory research, he recognized that he and his colleagues had made a category mistake in their treatment of objectivity by equating it with scholarly detachment from, and neutrality on, contentious political questions. While the latter was impossible to reconcile with commitment to emancipatory struggle, research could begin from the assumption that social phenomena have objective effects, while still committing to produce results which inform liberatory political strategies. No sooner was this point made, however, than Oliver dropped it—failing to draw out its implications for an alternative research practice.

I build on these insights by investigating what we might call the demand-side of EDR to evaluate how well it addresses problems in the research process for the disabled participants and activist groups it aims to recruit. I firstly recast EDR as a solution to a Credible Commitment Problem (CCP)—a control mechanism by which disabled stakeholders prevent researchers' deviating from the terms of their initial agreements with participants. When so viewed, previous accounts of EDR's failure to generalize across disability research, resting on the moral and professional preferences of those involved, are problematized. Inconsistent preferences can only be addressed by forcing parties to negotiate over which will be fulfilled and how in their collaborations. Stating that researchers hold preferences conflicting with EDR and act on these by spurning or sabotaging it is, therefore, a description of its limited growth, not an explanation for it.

In place of these, I argue that EDR failed to grow because it did not address a consistent preference of lay disabled people for actionable research outputs; based on assessments of objective processes in the social world instead of experiential responses to them, and using the scientific method to give confidence that emancipatory strategies could be built around their conclusions. I do not argue that this preference is universal. Clearly, some disabled people (not least the academics discussed in this article) do distrust the concept of objectivity, and value experiential research above all other kinds. I do argue, however, that a contrary preference is substantially and continuously expressed by activists and those sympathetic to their movement—limiting their incentives to enter negotiations over how research is conducted.

I evidence this, first, through the demand for scientific approaches to research proposed in the first critiques of research practice advanced within the Disabled People's Movement (Hunt, 2022a,1972,b). In these, I argue, the scientific method and a commitment to ontological realism are presented as integral to any meaningful negotiation over research practice: safeguarding research outcomes which lay participants can use in their other projects, and preventing both parties behaving inconsistently in the face of contradictory incentive structures. In the following section, I argue that this view is reflected in disabled participant and activist behavior during the period of EDR's emergence and propagation. Those projects conducted with EDR advocates which both participants and movement bodies recognized as successful forewent, in large part, EDR's emphasis on representing subjective experience. Similarly, the evidence we have of participant evaluation of projects which followed EDR's second and fifth principles more closely are largely ambivalent.

The literature on which this article is based comes, predominantly, from between the early-1970s and mid-2000s. This is, in large part, a reflection of the paucity of EDR studies produced in the last 20 years (as discussed in Section 3). While contemporary examples of EDR exist, and are discussed in Sections 2 and 5; these are a small minority within both the overall EDR literature and contemporary disability research.^3^ As I seek to explain EDR's failure to generalize since it's most productive period in the 1990s, this piece treats recent studies as tokens of their broader framework, rather than situating them in contemporary debates around co-research and expertise-by-experience^4^. This is, of course, something of a stylisation, but one (I hope) which allows us to draw insights on the strengths and weaknesses of EDR as a research project.

## 2 Disability research as credible commitment problem

Qualitative research requires the active consent of its subjects. Unless willing to limit their data to material already in the public sphere, or picked up through *ad hoc* observations, researchers must convince other human beings to answer questions, be observed, engage in odd experiments or research settings, etc. Participants might humor the researcher for any number of reasons—from an unselfish respect for science, to a (usually modest) payment for their inconvenience—but induced they must be. Clear information on the hows, whys, and whats of the research project are essential for establishing a moral economy^5^ in which consent can be given and maintained. A blossoming ethics and scientific integrity industry (including departments and committees at universities, academic publishers, and scientific associations) monitors the fairness of the resulting contracts between researchers and researched, and exacts costs on errant researchers. The odd scandal aside, the system works relatively well most of the time.

Inducements to participate in research are more problematic for members of marginalized and oppressed groups; particularly disabled people. Even bracketing the historical exploitation of, and violence toward, disabled subjects in the course of (social) scientific research^6^, many grounds which motivate others to engage in research may be irrelevant for disabled people, and the trust which mediates other research relationships may be lacking. The enlargement of science, or solution to general social problems, are likely less motivating for those whose marginality hinders them benefitting materially from either, and even cash payments can become the unwanted focus of scrutiny by benefits agencies to those trapped in long term unemployment (Rickard and Purtell, 2011, pp. 37–38). The benefits for researchers are clear—published qualitative research is a prerequisite for career advancement for many social scientists—but often indistinct (or simply more trouble than they're worth) for disabled subjects. This asymmetry gives the impression of exploitation, wherein the disabled researched give their time and effort for outcomes which will only payoff for the researcher. As one rehabilitation specialist has pointed out, the obvious question for participants remains: “what's in it for me?” (Amsters, 2019, p. 66).

This, combined with the proactive equation of marginality and vulnerability by university and funding bodies' ethics committees, incentivizes the researcher to offer a more comprehensive, and often more stringent, research contract to potential participants. It is likely that ethical problems and procedures—particularly those concerning participants' consent—will be defined in greater detail, with more responsibilities falling to the researcher to cultivate trust rather than simply avoid unethical activities. The proposed research outcomes are likely to be mooted as directly relevant to policy or cultural issues which disabled people are perceived by the researcher as having a stake in. Researchers might, further, promise to use conceptual frameworks sympathetic to the (real or perceived) interests and self-conceptions of disabled people; thereby turning research outputs into an authentic representation of participants' views, concerns, and experience.

While in many individual cases researchers succeed in offering a set of rules, topics, and analytic framework acceptable to enough people to make a given project viable, there are serious plausibility issues with each element of the expanded offer when stated abstractly. Thomas (2024) has recently noted the significant practical difficulties in enacting comprehensive research agreements in a reflection on a project he conducted with people with learning difficulties. The result of expanding the set of rules that a researcher must abide by is often a written research agreement so long and convoluted that it's unlikely that any participant (let alone one denied proper access to mainstream education) could fully familiarize themself with it—leaving the researcher and their professional colleagues the arbiters of compliance (pp. 11–12). If the agreement commits the researcher to activities which are particularly time intensive—such as allowing all participants to review analytic methods before results are written up—these may conflict with funders' strict timetables and are vulnerable to being ignored as the project progresses. This is particularly so when waiting for access adaptations to allow participants to complete these tasks stretches research timelines even further (pp. 19–20). Similarly, no matter how detailed the initial agreement, the researcher will still be faced with unexpected questions of power and consent during research practice. In disability research, these often arise from the actions of service providers whose presence is essential for the project to take place, but who may influence participants in ways which frustrate initial visions of consent and trust (pp. 15–17).

Even if these problems could be ameliorated, there are significant political contradictions in the research relationship which cannot easily be resolved. I, through Hunt (2022a,1972,b), will argue below that there is not just a power asymmetry between researcher and researched, but a structural opposition on who should receive the social and political authority associated with expertise. Disability is recognized by governments and civil society as an area requiring policy intervention, and those recognized as expert on it form a candidate pool for insider advisory positions (renumerated or otherwise) for the bodies which make and enact these interventions. Researchers are incentivized to keep this candidate pool small and credentialed to minimize competition, and to use research to prove their own suitability for it. Disabled people, on the other hand, are likely to want it radically widened to give them direct influence over decisions which affect their lives.

The research relationship, then, bears all the hallmarks of what economists and political scientists call a credible commitment problem (CCP) (North and Weingast, 1989, pp. 806–808). One party (in our case the researcher) is incentivized by the need to secure agreement to some action to make extensive commitments in the short-term which they either cannot, or will be incentivized not to, honor in the long-term. The leverage of other parties to control this behavior is time-limited, and will largely disappear once initial agreement is given. In our research context, once participants' data is collected, their leverage is largely spent; and ethics bodies are often incapable of solving disputes due to unclarity over how complaints should be made and adjudicated (Underhill, 2014, pp. 72–75).

Participants will, therefore, likely find themselves unable to enforce the terms of initial research agreements unless action is taken at the outset to limit which incentives the researcher can follow. As the problem emerges from inconsistencies between the researcher's preferences at different points in time, this must increase the dependency of researchers on participants (or at least a subset of them) throughout the research process: typically through constructing repeat interactions, formal arrangements which decentralize power away from the researcher, and mechanisms for monitoring compliance with research agreements which are independent of the researcher and their institution (Morriss and Ku, 2022, n.p.).

## 3 Emancipatory research and the CCP

As a solution to the CCP, emancipatory research should have performed well. It forces repeated interactions between researchers and disabled subjects (or those sharing their concerns), such that the researcher must consider the implications of their actions at later points in the project. It creates mutual dependencies between the researcher (who requires active consent for each project stage) and their subjects (for whom participation in project design creates greater interest in successful completion). That the DPM organized tens-of-thousands of disabled people at the time emancipatory research was proposed (Barnes, 1996b, p. xi) should have provided researchers with an extensive recruitment pool, and the DPM with the capacity to make use of research outcomes through political action. Theoretically speaking, EDR principles 1, 3, 4, and 6 put all the incentives in place for academics, disabled participants, and DPM actors to negotiate fruitfully over the form, content, and outputs of research practice.

Despite this, EDR failed to make much impact on disability research beyond its original progenitors; some of whom became disillusioned with it in the two decades following its initial flourish. Rix et al.'s (2020, pp. 1035–1037) study of participatory research with sensory and intellectually impaired people between 1996 and 2016 found only a small minority of projects which involved subject- or movement participation of long enough duration and high enough devolution to plausibly count as “emancipatory” (and even here this label was not necessarily appealed to). Their work further indicates that this is not because the CCP had been solved by other means. The comparatively small number of English, Spanish or German language studies discovered by them which involve disabled participants at all implies that incentivizing recruitment remains a significant general problem for disability researchers.

EDR's failure to generalize across disability research, despite solving a central problem of recruitment and consent, requires explanation. Those offered hitherto by both its proponents and detractors remain, however, unconvincing. Critics in the 2000s pointed to negative reactions to EDR within and without the academy, but without proving the relevance of these to its growth potential. Worth (2008, p. 311) suggested that academics might feel “intimidated” by the rigors of EDR's principles and prefer to avoid it. Certainly, both disabled and non-disabled academics experienced its initial proposal in this way: “a thinly veiled threat” to jeopardize non-compliant researchers (Bury, 1996, p. 113), or to impose stringent rules on those academics most likely to be disabled people, potentially curtailing their research careers (Shakespeare, 1996, p. 117). When emancipatory research is viewed as a response to the CCP, however, this appears as a feature, not a bug. Any solution to inconsistent commitments involves limiting researchers' ability to follow all their preferences through the application of leverage by other parties. This necessarily includes the threat of non-compliance or interference (i.e., by encouraging others not to participate in, fund, or disseminate particular projects). If participants and movement actors correctly judge their leverage, however, researchers should be compelled to enter negotiations despite their feelings of intimidation.

Similarly, Danieli and Woodhams's (2005, pp. 290–291) argument that EDR's growth was limited by the alienation of potential participants who aren't aligned with the DPM's theoretical and political commitments is something of a non-sequitur. There were, and are, plenty of disabled people who disagree more or less strongly with the DPM, but to prove that this impacted EDR's growth relative to other forms of disability research it must be shown that either:

a) this translates into a preference not to engage in emancipatory research compared to traditional, researcher-led projects;
b) the absence of participants who feel alienated could not be compensated by the pool of potential participants opened by movement actors' involvement in recruitment.

Danieli and Woodham offer evidence for neither claim.

EDR proponents, conversely, focussed their (often emotive) self-criticism on its inability to meet its 4^th^ principle of material benefit for disabled participants and empowerment opportunities for the movement—at least, relative to emancipatory researchers themselves. For Mike Oliver (1998, pp. 12-4), this resulted from researchers like him's failure to think beyond the researcher/researched distinction; leaving a hierarchical division of labor, and an unequal distribution of the benefits of research, unquestioned. This rationale is somewhat question-begging. If, as Oliver (1997, p. 188) holds, academics' ideological or “unconscious” biases toward this hierarchy are strong enough to jeopardize emancipatory aims; it must be explained why the participants and DPM actors the paradigm forced them to negotiate with were systematically unable to neutralize these subjective drives. Behavior based on ideological and epistemological commitments, or even the effects of the Id, are no less valid objects of negotiation for EDR than those arising from rational calculation. To say they caused research projects to diverge from their aims describes a process of failure (accurately or otherwise), without giving cogent reasons as to why divergence occurred.

More pragmatically, Gerry Zarb (1997, p. 50) pointed to the contradictory interests of funders and emancipatory movements to explain the impossibility of desired research outcomes. The DPM was antagonistic toward the state, the charity sector, and the medical establishment; yet these funded most disability research and would be unlikely to finance projects designed to undermine them. The funding reality at the time was more ambiguous than Zarb's neat explanation suggests. The entrance of charities and NGOs into research commissioning incubated, in many instances, a *laissez-faire* approach to project design (Mercer, 2004, p. 126). While plenty of funders rejected all radical projects, enough were prepared not to interfere in researcher-researched agreements for non-emancipatory researchers to plausibly fear (at least for a while) that EDR might become hegemonic (Bury, 1996, pp. 113–114; Danieli and Woodhams, 2005, pp. 291–292).

What these accounts lack is a reckoning with *whether* the potential participants and movement actors EDR was being offered to felt much need for it. It is assumed by all the accounts above that, if EDR could attract appropriate funding and stick steadfastly to its six core principles, it would unproblematically be perceived as a good, at least by those disabled people sympathetic to the DPM. In the next two sections, I outline significant evidence to the contrary. Disabled activists were very keen on controlling research practice; but far from enthusiastic about the commitments to reconstructing subjective experience and symbolic orders entailed by EDR's 2^nd^ and 5^th^ principles. Sympathetic non-activists, from the limited accounts we have of their evaluations, appear to value opportunities to share their unmediated experience with others, but to attach little value to the analytic and dissemination procedures EDR associated with this activity. Instead, we see (most clearly amongst activists and more ambiguously amongst other participants) a continuous preference for research into the economic, political, and social determinants of disablement, using the scientific method, which could inform disabled people's engagements with those phenomena.

## 4 Paul hunt: objectivity and the scientific method in early critiques of disability research

One of the earliest English-language critiques of academic-led disability research is Paul Hunt's sustained response to Miller and Gwynne's (1972) book on residential homes in Britain. Miller and Gwynne were invited by residents (including Hunt) at one such home in Surrey to investigate disputes between them, management, and staff over how much control residents should have over the rules and operational decisions of the home. Miller and Gwynne's conclusions had little bearing on the meat of these disputes. Instead, they argued that residential homes were the inevitable product of the economic parasitism and emotional dysfunction of disabled people, that their proper social function was to manage the transition between the social death caused by impairment and physical death, and that any tensions between staff and residents were best solved by a combination of psychoanalysis, euthanasia, and the imposition of military and colonial governance techniques.

Hunt's (2022a,1972,b) two responses to their work are unrelenting in their hostility and rigor. The second is generally seen by EDR proponents as the genesis of their own contribution to theorizing research practice: proving the demand amongst disabled people for a different way of structuring their relationship with researchers, and providing an analysis of pre-existing disability research as a form of exploitation (see, *inter alia*, Oliver, 1997, pp. 84–85; Mercer, 2002, p. 298; Stone and Priestley, 1996, pp. 702–703). From it, EDR took Hunt's claims that scholarly detachment and value neutrality were simply a screen to legitimate the biases and ideologies of elites and the pet theories of academics (Hunt, 2022b, p. 271); that academics were generally parasitic on the social problems of disability for their career opportunities (and thus uninterested in solving them) (p. 275); and that non-emancipatory research was primarily concerned with justifying the status quo rather than seeking reforms which would improve the lot of participants (p. 269).

Less appreciated (and, as we shall see, frequently contradicted) is Hunt's repeated appeal to the scientific method and norms of objective research; and the constitutive role these played in justifying other elements of his critique. In his initial retort to Miller and Gwynne, Hunt (2022a,1972, pp. 84–88) had complained not only that their work was dehumanizing, but also scientifically shoddy. They had generalized their conclusions from statistical outliers, designed interviews to solicit manipulable responses from participants, and selectively quoted other scholars to give the misleading impression that their assumptions were well-supported in their field. Hunt assumed that if these flaws were evident to him as a layman, they would lead other academics to discredit (or at least ignore) Miller & Gwynne's work. This assumption was proved false by *A Life Apart*'s growing influence throughout the 1970s (Hunt, 2022b, pp. 269–270).

In his second critique (2022b, 1981), Hunt's analysis of why this had happened and how disabled activists should respond was influenced by both his personal intellectual development, and by the changing balance of forces in British disability politics. Analytically, Hunt had spent much of the “70s deepening his engagement with Marxism. In Marx's (1971, pp. 498–522; 1991; pp. 956–957) critique of political economy, he distinguished between the scientific economists of the 17^th^ and 18^th^ Century, and the “vulgar,” unscientific economists who dominated in his own time. The former had aimed at accurate descriptions of capitalism's workings and rigorous explanations of its social effects. The latter's' role Marx characterized as developing increasingly implausible apologies for capitalism's brutality, and solving trivial efficiency problems for one or another branch of industry.

For Marx, the transition between the two rested on the subsumption of intellectual endeavor to the social relations of capital at the end of the 18^th^ Century. As the bourgeoisie took over the patronage functions of the old aristocracy, they simultaneously assumed control of how research was paid for (by retainer or university employment), how it was disseminated (publishing), and the terms of access to its necessary materials (records, workplaces, etc). To access these resources, intellectuals must prove themselves useful *to* this bourgeoisie and, importantly, avoid uncovering any unpleasant truths that might impede future investment (such as capital's crisis tendencies). The objective social world investigated by previous economists had not disappeared, Marx argued—and nor had their methods of investigation become obsolete. The social relations of intellectual life had, however, now altered such that this world could not be honestly approached, nor these methods fearlessly used, within the institutions and cultures of intellectual life. Scientific practice could be advanced only by those who decouple their research from the authoritarianism of intellectual milieus; taking their impetus from workers' movements who have no desire to make excuses for the present order, and thus free to face it without distortion.

Hunt (2022b) developed an analogous argument for disability research. Miller and Gwynne had not only agreed the terms for their research with government funders; but, by virtue of undertaking their project, had entered a highly competitive market of “experts” qualified to advise the state and service providers on the management of disability services and policy transitions. States and providers have strong policy commitments, based on their previous practice and the distribution of power within them. If the scientist tells them what they want to hear, or provides recommendations which they would like to implement; the rewards can be lucrative. If the scientist discovers inconvenient facts (that these policy preferences are falsely premised and dishonestly justified), they're likely to exit the market promptly (pp. 277–278).

It was pandering to such preferences at the expense of scientific investigation, in Hunt's estimation, that led Miller and Gwynne to accept that physical impairment caused social irrelevance, and that authoritarian segregated services were its necessary corollary (p. 274). By contrast, disabled people themselves have no such material incentives to inhibit scientific practice:

“Faced with any socially oppressed group, social scientists have a choice of only two alternatives: either a firm commitment to serve the interests of the oppressed group to end their oppression, or a commitment to serve the interests of the oppressors to continue their oppressive practices (which last they also do by serving their own interests). There can be no middle way.

In the first instance a scientific approach remains possible, i.e., objective reality can be looked at, and science can be placed at the service of the oppressed group to help them free themselves. In the latter instance a scientific approach is not possible, objective reality cannot be examined straight but can only be distorted. (…) It is commonly believed that commitment to the cause of an oppressed group means that “reality” will be ignored or distorted, and therefore that the best scientist is the one who tries to be least involved and most detached. Nothing could be further from the truth, as *A Life Apart* illustrates. It is precisely those who try to take a detached view of oppression who cannot be objective.” (p. 275)

The political factor which influenced Hunt's account was the emergence of an independent DPM: capable of taking advantage of disabled people's ability to view the world objectively by aggregating collective experience to direct research, evaluating its processes and findings, and acting on its conclusions. Where Miller and Gwynne rejected calls to objectivity (claiming that social science should aim only at improving efficiency, not a true explanation of the world) (in ibid, p. 272), this movement was:

“enabled to view reality objectively, recognizing the potential [for liberation] that has now been made possible and by contrast the oppressive conditions of life that we are forced to put up with. The important thing is that our approach maintains a scientific analysis of our situation, which examines segregated institutions objectively within the context of modern social developments, [and which] is both necessary and possible.” (p. 268).

Hunt's conception of what this “scientific analysis” consists in was fairly traditional in its philosophy. Hunt offered two core principles for the kinds of research practice that the DPM should demand: Firstly, all investigations should accept external criteria of falsification^7^ and evaluation. Any conclusion reached by (academics' or disabled people's) intellectual practice should be open to contradiction by lay disabled people's observation of the world around them. With this principle, disabled people could test the legitimacy of research, and establish whether its findings were solid enough to inform their political strategies. Without it, intellectual pursuits would be “about as scientific as magic” (p. 272).

The second, only slightly more ambitious (and influenced again by Hunt's Marxism) was that research must capture the relationships between the material effects of social phenomena in a state of flux and tension. Disabled people's emancipation projects occur in a changing social world, characterized by economic and political struggles, and where actions are liable to lead to unexpected consequences when they are not informed by the most nuanced analysis possible. The variables necessary for such an analysis are not captured by the limited experiential standpoints occupied by the members of an oppressed group. The function of science, for Hunt, is to uncover the determinants of disablement which elide the lived experience of being disabled:

“Oppressed groups have nothing to lose, and everything to gain, from the most precise and thorough understanding of the situation we are struggling to change. To change our oppressive reality, we cannot afford to leave out of account any significant factor in the situation: to do so necessarily means defeat (…) A scientific approach must look at a part in relation to the whole, or institutions in relation to the society in which they exist. It must look at social forces as in a state of movement and development, not as being static; and, therefore, it must look at institutions in the context of a changing society. It must also look at the struggles of people for change in relation to the material and social changes that have taken place in the society, not as mere reactions to irreversible natural causes” (p. 276).

## 5 Emancipatory research and the turn to subjective experience

If Hunt had been “pioneering” in his analysis of the tensions between disability researchers and disabled participants (Oliver, 1997, p. 84); his proposals to reform this relationship were less convincing. Hunt recognized that academic researchers had skills which the movement needed as well as perverse incentives, but died suddenly before finishing his second critique. He left a draft questionnaire (2022b, 1981, pp. 282–4), which he had expressed grave reservations about to his comrades, to gauge the political commitments of any academic approaching the movement for research participants. This prescription was manifestly insufficient given the diagnosis he had made. Hunt had shown that researchers' incentives and preferences were likely to change throughout a research process, but his solution was merely to filter some explicit preferences out at its earliest stages.

Later emancipatory researchers attempted to correct this gap between analysis and action, and develop “a methodology and set of techniques commensurate with the emancipatory research paradigm” (Oliver, 1992, p. 112). It is in their work of the 1990s that the technical innovations most conducive to solving the CCP are theorized, implemented, and reported as examples for future practice. Operational suggestions are advanced to decentralize control of researcher decisions across project stages—from design, to data collection, to evaluation (Priestly, 1997); formative evaluation criteria are formulated for participants to assess if a project is amenable to their control (Zarb, 1992, pp. 128–129); and multiple strategies for user-direction are developed for instances where funding arrangements and logistics prevent stakeholders' direct supervision of the researcher (summarized in Mercer, 2004). This infrastructure should have had a positive effect on bringing participants and researchers together. The framework developed in the literature did a great deal to align the expectations of participants (who could see what the research process might entail and their leverage in it) and researchers (who were given clarity on their obligations in EDR).

Simultaneously, these scholars altered profoundly the analysis on which the initial demand for research had been made. Hunt (2022b, p. 272) had argued that it was disablist “bias” which leads “to a project totally lacking scientific validity.” EDR proponents claimed the exact opposite: that even the pretense of scientific validity, and the realist ontology on which it rests, directly caused disabled people's dehumanization—sometimes citing unrelated passages from Hunt to justify this claim^8^. Instead of a great leveler, which allowed disabled people to evaluate and act on research findings, claims to objectivity were seen as no more than an “ideology” (Zarb, 1992, p. 130); “falsely premised” on oppressive social relations (Priestly, 1997, p. 90), and invariably justifying the right of a “relatively small group of powerful experts [to] work on a larger number of relatively powerless research subjects” (Oliver, 1992, p. 106).

The fundamental problem with previous disability research was seen not as its failure to increase disabled people's understanding and capacity for action; but that its descriptions alienated them from their senses of self and distorted their life-experience. The emancipatory response was to proclaim fidelity to both: focussing on the “symbolic world in which the subject lives” (Barnes, 1992, p. 116) and the “meaning of events [from participants” perspectives] not their causes' (Oliver, 1992, p. 106).

There is little evidence that this focus on subjective meaning-making was much in demand: either by organizations in the DPM, nor disabled lay people who become research participants. The research projects commissioned, managed, and distributed by movement organizations indicate a demand for research practice which mirrors Hunt's insistences on analyses of social processes and principles of external falsification (albeit absent his Marxist meta-theory). Despite its author's later claim that it constituted the paradigmatic instance of EDR (Barnes, 2004, n.p.); Barnes's (1991) study of discrimination in Britain (commissioned and supervised by a national DPO) shows little sign of abandoning objectivity or causality, nor reconstructing obscured subjectivities. Instead, the movement instructed Barnes to evidence discriminatory institutional practices across various social spheres (education, employment, leisure, etc), determine the causes of these practices, and deduce the material impacts of proposed or actual government policies on them (p.62).

Other movement-managed research shows a similar orientation toward rigorously examining impersonal social causes. Movement organizations commissioned Macfarlane and Laurie (1996) to examine the relationship between deinstitutionalisation policies and the provision of accessible housing, and Zarb and Nadash (1994) to determine the likely costs of the DPM's proposals for community support relative to existing forms of “community care”. Those instances where movement organizations allowed their researchers to deal more substantively with the personal experience of disablement are outliers, and justified by specific project aims rather than the inherent value of personal standpoints as a source of knowledge. Oliver et al.'s (1988) extensive interviews with spinally injured people, for example, responded to the extreme variation in services and living situations around the country, and the fact that “[t]here was little prior work on which to build”. Considering this, in-depth discussions of personal experience were the most reliable source of objective and quantitative, as well as qualitative, data (pp. 7–8). It is clear from this engagement that DPM actors were keen to work with emancipatory researchers, and took full advantage of the opportunity to control more of the research processes, but encouraged EDR practitioners to leave their anti-objectivity commitments at the door to pursue knowledge that the movement couldn't source from within its own ranks^9^.

EDR practitioners have, regrettably, seldom reported evaluations of their practice by disabled participants outside of movement organizations. Where they have, however, participants appear to be largely ambivalent on the value of reproducing their unalienated experience for academic papers or research reports. Gabutt and Seymour (1998, pp. 8–9) report that, in a project where participants were asked to use their personal experience of disablement to code interviews with professionals, participants were initially keen to talk together about their life histories and personal responses to the data. As the project progressed, however, roughly four-fifths of the participants were disengaged at any one time; with one participant expressing doubt that the project's focus displayed “the will to bring about change” (p. 9). While representing their own life experiences to their peers appears to have been a self-motivating good for most participants, the promise of a researcher faithfully reproducing it again for others was insufficient to secure their long-term collaboration.

One of the rare recent projects to invoke EDR as a paradigm (Liddiard et al., 2019) provides further evidence, in the form of a dog which refuses to bark, of participants' limited demand for researchers reconstructing their identities and experience. The academics working on the project began from the theoretical commitment that disabled people are “DisHuman”—complex assemblages of bodily and phenomenological states which elide and reject distinctions between humans, animals, and technology (p. 1049). They soon discovered, however, that lay “co-researchers” strongly believed themselves to be human beings. This was explained as an understandable life-strategy reflecting participants' marginalized position. If societies ascribe status and recognition to those categorized as human, it is natural to claim membership of this category when one is afforded neither. Such claims, however, were analytically secondary. The fact that *some* data about participants could be interpreted as consistent with the DisHuman thesis justified its continued deployment in theoretical descriptions of their lives. Put bluntly, the academics knew better than the lay-person how to analyse the latter's identity, up to and including ascribing their species. Participants were DisHuman regardless of whether they considered themselves so (pp. 1050–1051). The fact that participants neither insisted on reversing this conclusion through available negotiating mechanisms^10^, nor withdrew if this proved impossible, implies that they didn't require the research to validate or faithfully represent their experience. Clearly, participants saw something of value in their continued engagement, and tolerated alien descriptions of their lived experience in pursuit of it.

## 6 Conclusion: re-emancipating disability research

The hypothesis I have offered is that EDR failed to either generalize across disability research, nor contribute consistently to improving disabled people's lives, because it's focus on subjective experience and rejection of objectivity and the scientific method clashed with what a sizeable number of disabled people wanted research to do. I have evidenced this by outlining a sustained demand by movement actors for rigorous research on social phenomena that cannot be reduced to subjective meaning-making, and by indicating ambivalence toward EDR's research focus from participants more widely. By reframing EDR as a solution to non-credible academic commitments, I have problematised other explanations of the same phenomenon; indicating that these merely describe EDR's underwhelming progress rather than identify its causes.

I have, hitherto, avoided giving anything like a positionality statement. Like Hunt and Marx, I suspect that personalized data-points are the least useful for rigorous argumentation. As I believe I have shown that my position on research is not wholly idiosyncratic, and in the hope that the observations which spurred this argument might also be relevant to thinking about research differently, I offer the following as a coda.

In addition to my academic research in disability history—supervised by a Steering Group within a movement organization—I hold positions of responsibility in two Disabled People's Organizations (DPOs) at the time or writing, and have previously been commissioned to run a research project at another. I and my comrades are frequently approached (usually by keen PhD students) to become partners on research projects. While the level of control offered to us varies, the emphasis on reproducing the authentic voice of the disabled people we work with is pretty constant across these approaches.

I have two concerns whenever such research is mooted. The first is skepticism that it will tell us anything we don't already know, or provide our members with something they don't already own. We are in touch with the same people the researcher is asking us to facilitate access to. If we need to ask their experience of something (and they've likely told us their views forcefully already), we can do so without the aid of intermediaries. Similarly, members of our networks can already represent their own experience and identities at very low cost. Blog posts and social media profiles, and before them movement “zines and newspapers, allow disabled people to say whatever they want to an audience larger than most academic journals” readership. Experience and voice, like culture, are things people already have and cannot be given to them. It flatters no-one to make a virtue of wrapping them up as if they were a gift (Sivanandan, 2005, n.p.)^11^.

The second is logistical. Entering partnership on a research project diverts a lot of organizational resources. At bare minimum, we will have to assign one member to read and comment on extensive drafts, cultivate enough knowledge of the subject area to properly monitor the researcher's practice, and condense the research content and progress to report back to other members. Depending on the research, the actual commitment could be much greater. Our member is constrained from taking part in all the other work the DPO needs them to do while the research is ongoing, and the rest of us must divide their share amongst ourselves. This is a sacrifice worth making *if* the research is likely to give us information we need to further the liberation struggle, or if the process will help our member develop research skills we can use for other purposes. If it doesn't, it is simply a bad investment.

These concerns can be addressed by making emancipatory research about disablement—the economic, social, institutional, and environmental factors which shape the lives of people with impairments, mental distress, or neurological difference—rather than some aspect of disabled people themselves. Realistically, any impetus toward this must, in the short term, come from the academy. The DPM (in Britain at least) is small and cash poor. It is in no position to commission large research projects in line with its needs, nor to exert the same leverage in negotiations with researchers that it could in the ‘90s and 2000s—which likely makes some organizations reticent about agreeing to research partnerships. If we are to save what is good from EDR—its emphasis on empowering subjects and its democratization of research practice—it is necessary for the academics who pitch most disability research to attend to the external factors which prevent disabled people from enjoying the same freedoms as their peers.

## Footnotes

^1^For the purposes of this article, I use ‘DPM' to refer to the aggregation of self-organised networks or groups of disabled people involved in political work (broadly understood to include protest action, politically informed models of self-empowerment and service provision, etc). I bracket here, for the sake of simplicity, the question of whether ideological, social or organisational distinctions between such networks signify multiple distinct movements, or a single movement with multiple tendencies. ‘The DPM', in singular, will accordingly be used throughout the rest of this piece.

^2^For early statements of this critique see, *inter alia*, Morris, 1989 (esp. pp. 5–7); 1992; Oliver, 1992; Zarb, 1992; Barnes, 1992; Abberley, 1993.

^3^In contemporary disability research in the Global South, “emancipatory disability research” is often used to describe a much looser framework than the one I interrogate here. This approach prioritizes participants' skill development over their control of research practice—by, for example, using research projects to build networks between participants from different communities (Cutajar and Adjoe, 2016, p. 506), or training local disabled people to conduct research under academic supervision (Deepak, 2015, pp. 6-7).

^4^For an overview of such debates, see Dembele et al., 2024.

^5^I, following Thompson (1971), use “moral economy” to refer to both relatively stable mores or customs which align the behavior of one party with the expectations of another, and shared moral commitments which allow the recognition an activity's aims and value to converge across social positions. In the case of research, the latter might include: the enlargement of knowledge; the practical solution of some (social/medical/practical) problem; or the obligations of all parties to abide by the terms of a Participant Agreement. For an exegesis of Thompson's concept, see Edelman (2012, pp. 55–58).

^6^For the alternative view, that contemporary disability research cannot be separated from the most abusive historical forms of research practice (particularly those undertaken in the Third Reich), see Sierck and Radtke, 1984; Pfeiffer, 1994; Mostert, 2002.

^7^The word Hunt uses here is “verification”; reflecting lay understandings of the philosophy of science current when he was writing. I have altered the term here as the passage I paraphrase is concerned with disabled people's ability to falsify, and thus dispense with, incorrect conclusions.

^8^See, *inter alia:* Stone and Priestley, 1996, pp. 702–703; Priestly, 1997, p. 91; Barnes and Sheridan, 2007, pp. 239–40.

^9^There is some evidence that this preference is not exclusive to the British DPM. A review of disability research across Southern Africa commissioned by the Southern African Federation of the Disabled concluded that the most immediate task for academics and DPOs was to encourage research outputs that could be acted on by disabled activists (Mckenzie et al., 2014, pp. 740–42).

^10^The project had a formal participants' council, although its exact powers are unclear (Liddiard et al., 2019, p. 1038).

^11^While a detailed analysis of Feminist Disability Studies is out of this article's scope, my argument here suggests that some of its premises may be ill-conceived. If the role of feminist disability research is to ‘allow space (…) for the absent subject' (Morris, 1992, p. 159), or ‘retrieve dismissed voices' (Garland-Thomson, 2005, p. 1557); the theorist must explain the productive role of academic research in ending this marginality. My contention is that the dismissed may not be so absent as is asserted, and may have more powerful tools at their disposal than those offered by academicians. For an alternative view of the relationship between feminist and disability politics (and consequently research), see Rae, 1996.
